# Lead Bullet Fragments in Venison from Rifle-Killed Deer: Potential for Human Dietary Exposure

**DOI:** 10.1371/journal.pone.0005330

**Published:** 2009-04-24

**Authors:** W. Grainger Hunt, Richard T. Watson, J. Lindsay Oaks, Chris N. Parish, Kurt K. Burnham, Russell L. Tucker, James R. Belthoff, Garret Hart

**Affiliations:** 1 The Peregrine Fund, Boise, Idaho, United States of America; 2 Washington Animal Disease Diagnostic Laboratory, Pullman, Washington, United States of America; 3 Department of Veterinary Clinical Sciences, Washington State University, Pullman, Washington, United States of America; 4 Department of Biology, Boise State University, Boise, Idaho, United States of America; 5 School of Earth & Environmental Sciences, Washington State University, Pullman, Washington, United States of America; East Carolina University, United States of America

## Abstract

Human consumers of wildlife killed with lead ammunition may be exposed to health risks associated with lead ingestion. This hypothesis is based on published studies showing elevated blood lead concentrations in subsistence hunter populations, retention of ammunition residues in the tissues of hunter-killed animals, and systemic, cognitive, and behavioral disorders associated with human lead body burdens once considered safe. Our objective was to determine the incidence and bioavailability of lead bullet fragments in hunter-killed venison, a widely-eaten food among hunters and their families. We radiographed 30 eviscerated carcasses of White-tailed Deer (*Odocoileus virginianus*) shot by hunters with standard lead-core, copper-jacketed bullets under normal hunting conditions. All carcasses showed metal fragments (geometric mean = 136 fragments, range = 15–409) and widespread fragment dispersion. We took each carcass to a separate meat processor and fluoroscopically scanned the resulting meat packages; fluoroscopy revealed metal fragments in the ground meat packages of 24 (80%) of the 30 deer; 32% of 234 ground meat packages contained at least one fragment. Fragments were identified as lead by ICP in 93% of 27 samples. Isotope ratios of lead in meat matched the ratios of bullets, and differed from background lead in bone. We fed fragment-containing venison to four pigs to test bioavailability; four controls received venison without fragments from the same deer. Mean blood lead concentrations in pigs peaked at 2.29 µg/dL (maximum 3.8 µg/dL) 2 days following ingestion of fragment-containing venison, significantly higher than the 0.63 µg/dL averaged by controls. We conclude that people risk exposure to bioavailable lead from bullet fragments when they eat venison from deer killed with standard lead-based rifle bullets and processed under normal procedures. At risk in the U.S. are some ten million hunters, their families, and low-income beneficiaries of venison donations.

## Introduction

Lead has been impacting the health of humankind since the Romans began mining it 2500 years ago, and despite early knowledge of its harmful effects, exposure to lead from a wide variety of sources persists to this day [1]. Government-based guidelines for acceptable degrees of exposure prior to the 1970s were based upon thresholds of overt toxicity and on apparent acceptance that norms in lead concentrations in a society enveloped in lead-permeated exhaust fumes and lead paint must somehow reflect organic tolerance. Medical science has since concluded that virtually no level of lead exposure can be considered harmless in consideration of its many sublethal, debilitating, and often irreversible effects [2]. Lead quantities formerly regarded as trivial are associated with permanent cognitive damage in children [3], including those prenatally exposed [4]. Lead is associated with impaired motor function [5], attentional dysfunction [6], and even criminal behavior [7], [8]. Release of lead stores from bone exposes fetuses during pregnancy [9], and adults late in life [10], [11]. Lead is implicated in reduced somatic growth [12], decreased brain volume [5], spontaneous abortion [13], nephropathy [14], cancer, and cardiovascular disease [15], [16].

Ingested residues of lead ammunition are a recently identified pathway of lead exposure to human consumers of gun-killed game animals. An analysis of North Dakota residents showed that recent (≤1 mo) consumers of game meat had higher covariate-adjusted blood lead concentrations than those with a longer interval (>6 mo) since last consumption [17]. Studies have linked elevated blood lead concentrations of subsistence hunters in northern Canada, Alaska, Greenland, and elsewhere to consumption of shotgun-killed birds [18]–[25; see 26,27]. The hypothesis that rifle bullet fragments are an additional source of human lead exposure is suggested by radiographic studies of deer killed with standard lead-based bullets, which show hundreds of small metal fragments widely dispersed around wound channels [28]–[30]. The possibility of inadvertent lead contamination in prepared meat consumed by hunters and their families is noteworthy, considering the millions of people who hunt big game in the U.S. [31] and the thousands of deer annually donated to food pantries for the poor [32], [33]. In this report, we test two hypotheses: (1) that fragments of lead from rifle-bullets remain in commercially processed venison obtained under normal hunting conditions in the U.S., and (2) humans absorb lead when they eat venison containing bullet fragments.

## Materials and Methods

### Ethics statement

Nine licensed hunters provided the deer carcasses analyzed in this study, and obtained them during the established hunting season and in accordance with normal practices as permitted under the authority of the Wyoming Game and Fish Commission, Cheyenne, Wyoming. The latter institution also granted permission to the authors to convey the processed meat from each carcass to the Washington Animal Disease Diagnostic Laboratory at Washington State University, Pullman, for analysis. The Washington State University Institutional Animal Care and Use Committee approved the lead bioavailability experiment involving eight swine.

### Deer collection

Hunters used conventional center-fire hunting rifles to kill 30 white-tailed deer (*Odocoileus virginianus*) under normal hunting conditions in Sheridan County, Wyoming in November 2007. All bullets were of 7-mm Remington Magnum caliber and of identical mass (150 grains, 9720 mg); cartridges were of a single brand reported in local mass-market vendor interviews as the most widely sold to deer hunters. Bullets consisted of a lead core (68% of mass) and a copper jacket (32%); lead was exposed only at the 1.7-mm-diameter tip of the bullet. Reported shot distances averaged 116 m (range = 25–172 m). All deer were eviscerated according to the hunters' normal practice. Weights of 29 eviscerated deer averaged 33.8 kg (SD = 7.1). We recorded the positions of bullet entry and exit wounds; 26 deer (87%) were shot in the thorax, and some portion of the projectile exited the animal in 92% of shots. We removed the skin and head, and we excised from each animal a ≥4 cm section of tibia for isotope analyses and a ≥30 g sample of muscle (shank) along the tibia to determine background lead levels in each deer.

### Carcass radiography

We radiographed with conventional veterinary equipment the area of the wound channel (lateral view) of eviscerated deer and adjusted exposures to maximize contrast. We included along the margin of each radiograph a strip of clear plastic tape containing arrayed samples of lead bullet fragments (obtained by shooting through light plastic jugs filled with water), comparably-sized samples of bone fragments, and locally-obtained sand and gravel; only the lead fragments were clearly visible in the radiographs at the applied settings. We scanned radiographs into digital format and counted unambiguous metal fragments under 400% magnification. We did not attempt to distinguish between copper and lead in fragment counts.

### Commercial processing

We transported each deer carcass to a different commercial meat processing plant in 22 towns throughout Wyoming and requested normal processing into boneless steaks and ground meat in 2-pound (0.91 kg) packages; we retrieved the processed, frozen, and packaged meat usually within 4 days.

### Radiography of processed meat

We used digital radiography (EDR6 Digital Radiography, Eklin Medical Systems, Santa Clara, California) and fluoroscopy (MD3 Digital Fluoroscopy, Philips Medical Systems, Best, Netherlands) to scan all the thawed ground meat packages (N = 234); we scanned an additional 49 loin steak packages from 16 carcasses in which radiography had revealed fragments near the spine. We unwrapped every package showing visible radiodense fragments in a subsample of 13 deer, flattened the meat to c. 1-cm thickness on a light plastic plate, and rescanned. We marked the vicinity of each visible fragment with a stainless steel needle and then used a 2.8-cm diameter plastic tube as a “cookie-cutter” to obtain samples of meat with radiodense fragments.

### Analysis of metal samples

Each of the fragment-containing meat samples was weighed and then divided into approximately 5-g subsamples, each of which was completely digested in a known volume of concentrated nitric acid. Inductively coupled plasma (ICP) analysis was then used to measure the concentrations of lead and copper in each subsample. The lower detection limit for both metals was 2 µg/g. The analysis was performed commercially by the Analytical Sciences Laboratory, University of Idaho, Moscow, where quality management conforms with applicable Federal Good Laboratory Practices (40 CFR Part 160); the Laboratory is accredited through the American Association of Veterinary Laboratory Diagnosticians, which stipulates ISO 17025 quality assurance measures.

### Lead isotope analysis

We analyzed bullet, bone, and meat samples for lead isotope compositions. Bullet fragments were cleaned in dilute (1 M) HCl, leached with 2 ml of 7 M HNO_3_, and then removed from the acid leachate. The leachate was then dried and treated with 2 drops of 14 M HNO_3_. Bone and meat samples were digested in 14 M HNO_3_, dried and treated with 2 drops of 14 M HNO_3_. Lead was separated using standard HBr and HCl on an anion-exchange column (Bio Rad, AG 1×8). Isotope compositions were determined with a ThermoFinnigan Neptune MC-ICPMS at the Washington State University GeoAnalytical Laboratory. Reproducibility of the lead standard (NBS-981), run before, during, and after the samples, was <0.012% (2 SE, n = 4) for 206Pb/204Pb, and <0.018% for 208Pb/204Pb. Lead concentrations in the procedural blanks were negligibly small.

### Bioavailability experiment

We tested the bioavailability of ingested bullet fragments by feeding processed venison known by radiography to contain radiodense fragments to pigs. The latter were considered a good model for the absorption of lead from the human gastrointestinal tract [34]. We used eight female Yorkshire/Landrace and Berkshire/Duroc cross-bred pigs, 70–82 days of age and weighing 28.2–32.7 kg (mean 30.3 kg) at the termination of the experiment. All were initially fed 1.36 kg of standard pelleted pig grower ration divided into two meals per day, then acclimated for 7 days to consuming cooked ground commercial beef patties mixed with the pellet ration. We gradually increased the amount of ground meat from 113 g per meal to 500 g, as pellet amounts were correspondingly decreased. We withheld all food for 24 hours prior to the venison feeding trial.

Ground venison and venison steaks from four deer were used in the feeding trial. Each of the eight pigs consumed 1.26–1.54 kg of meat over two feedings 24 hours apart on days 0 and 1 of the experiment; no pig consumed meat from more than one deer. Four pigs received venison containing fluoroscopically visible metal fragments. The total amount of lead fed to each pig was unknown, but quantitative analysis of similar packages from other deer in the study showed 0.2–168 mg (median 4.2 mg) of lead. The four control pigs were simultaneously fed equivalent amounts of venison with no fluoroscopically visible fragments from the same four deer. We assessed background levels of lead in each deer from shank meat, collected well away from any potential bullet contamination. All venison for the test and control pigs was either already ground, or finely chopped if steaks, and cooked in a microwave oven until brown. For feeding, we mixed the cooked venison in a bowl with small amounts of pig ration to improve palatability. We verified that all meat was eaten, and we monitored the pigs for signs of illness.

We collected anticoagulated blood samples (2 ml whole blood in EDTA) from each pig at 1 hour prior to feeding venison on day 0, and on days 1, 2, 3, 4, 7 and 9 after feeding venison, and stored the samples at 4°C until testing. Lead levels were determined by inductively coupled plasma mass spectrometry (ICP-MS) with a lower detection limit of 0.5 µg/dL; we assigned all values below the detection limits as 0.5 µg/dL. We compared mean blood lead concentrations between control pigs and test pigs on days 0 through 9 using 2-way ANOVA with repeated measures and restricted maximum likelihood (REML) estimation; we performed linear group contrasts for each day. A single outlier datum among control pigs on day 4 (6.8 µg/dL) was an order of magnitude higher than a retest of the same sample (0.54 µg/dL); the latter was consistent with all other control samples. We omitted both results from statistical analysis, resulting in a sample of three rather than four control pigs on day 4. We used JMP (SAS Institute, Cary, NC, USA, Vers. 7.0.1) for all statistical analyses.

## Results

### Bullet fragments in venison

Wound radiographs of all 30 eviscerated deer showed metal fragments (median = 136 fragments, range = 15–409) and offered a measure of fragment dispersion, albeit two-dimensional. Extreme distance between fragment clusters in standard radiographs averaged 24 cm (range±SD = 5–43±9 cm), and maximum single fragment separation was 45 cm. Radiography revealed visible metal fragments in the ground meat of 24 (80%) of the 30 deer. At least one fragment was visible in radiographs of 74 (32%) of 234 packages of ground meat; 160 (68%) revealed no fragments, 46 (20%) had one, 16 (7%) had two, and 12 (5%) showed 3–8 fragments. An average of 32% of ground meat packages (N = 3–15 packages, mean 7.8) per deer showed metal fragments (range = 0–100% of packages). The ground meat derived from one deer showed more fragments (N = 42) than counted in the radiograph of the carcass (N = 31), and two ground meat packages (2 deer) each contained a single shotgun pellet which had not been detected on the carcass radiographs. No relationship was apparent between the number of metal fragments counted in carcasses and those subsequently counted in ground meat from the same individual (correlation coefficient 0.06). In the aggregate, we observed 155 metal particles in the ground meat packages, 3.1% of the 5074 we counted in the carcasses. Of 16 deer carcasses with metal fragments near the spine, four (25% of selected deer, 8% of 49 packages) showed fragments in processed loin steaks (1–9 fragments). Additional fragments may have occurred in 220 unscanned packages of steaks derived from all animals.

ICP analysis of radiodense fragments excised from ground meat packages from 13 deer identified lead in 25 (93%) of 27 samples; aggregate lead fragment mass per package averaged 17.2 mg (range±SD = 0.2–168±39.8 mg) or 0.03% of the lead component of bullet mass. Nine samples contained copper at levels above background values, including the two samples with no detectable lead. Lead concentrations in unprocessed muscle tissue collected from the shank and well away from the bullet path of the same 13 deer were all below the detection limit of 2.0 µg/g and served as internal controls for measures of lead in ground meat.

**Figure 1:**
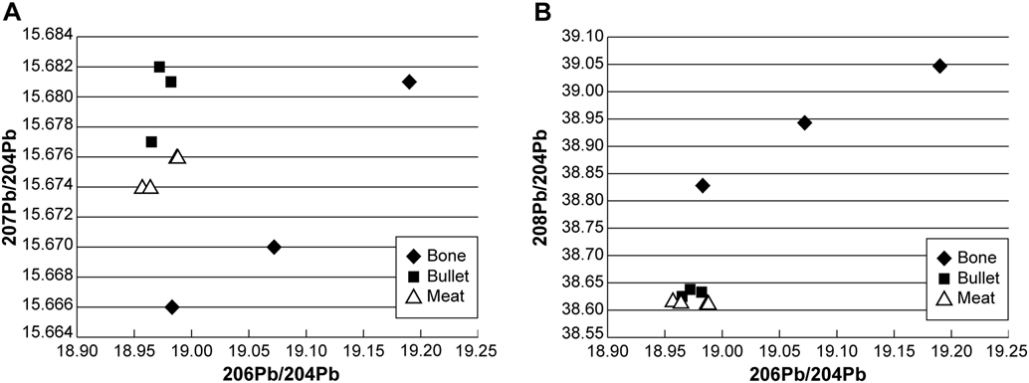
Plots of lead isotope ratios in ground meat samples containing radiodense fragments from four deer. Ratios from lead-in-meat samples clustered with those of unfired bullets but were distinct from bone lead ratios. Note that there are four meat data points (open triangles) in each graph, but two have almost identical positions and are superimposed.

The ratio of lead isotopes 206/204 plotted against 207/204 ratios (Fig. 1(a)) and 208/204 ratios (Fig. 1(b)) showed that meat samples with elevated lead levels from four deer, and lead from bullets from the same boxes (N = 3) supplying the bullets used to kill those deer, formed tight clusters distinct from ratios of background lead in tibial bone. Variation in the bone ratios apparent in Fig. 1 likely represent long term, cumulative lead exposure encompassing varied sources of natural and anthropogenic lead.

### Bioavailability experiment

All the pigs consumed all the venison provided to them within 2 hours. None of the experimental animals showed any signs of lead toxicosis or other illness for the duration of the experiment; none exhibited vomiting or diarrhea which might have affected gastrointestinal physiology or retention times in the stomach or intestines.

**Figure 2:**
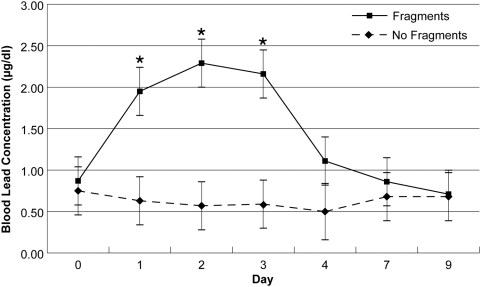
Mean blood lead concentrations observed during swine feeding experiment. Mean (±SE) blood lead concentrations (µg/dL) in four pigs fed venison containing radiographically dense fragments (Fragments) compared with four control pigs fed venison without visible fragments (No Fragments) on days 0 and 1. Asterisks indicate days when means differed significantly between test and control groups.

Blood lead concentrations in the four control pigs ranged from below the level of ICP-MS detection (0.5 µg/dL) to 1.2 µg/dL throughout the experiment (mean±SD = 0.63±0.19 µg/dL; Fig. 2). Blood lead concentrations in pigs fed metal fragment-containing venison ranged from below the level of detection to 1.4 µg/dL on day 0, immediately prior to feeding venison. The 2-way ANOVA revealed a significant interaction between treatment (feeding venison either with fragments or no fragments) and day (F_6,35.32_ = 3.413, P = 0.009; Fig. 2). Mean blood lead concentrations in the pigs fed fragment-containing venison were significantly elevated above those of control pigs on days 1, 2 and 3 post-exposure (linear contrast: F_1,39.79_ = 10.39, P = 0.003, F_1,39.79_ = 17.76, P = 0.0001, and F_1,39.79_ = 14.71, P = 0.0004, respectively; Fig. 2); the maximum observed value was 3.8 µg/dL. Blood lead concentrations did not differ (P>0.05) between the control pigs and exposed pigs on days 0, 4, 7 and 9 (Fig. 2).

## Discussion

Our findings show that people risk exposure to bioavailable lead when they eat venison from deer killed with standard lead-based rifle bullets and processed under normal commercial procedures. Evidence includes a high proportion (80%) of deer showing at least one bullet fragment in one or more ground meat packages, a substantial frequency of contamination (32% of all ground meat packages), a majority (93%) of assayed fragments identified as lead, isotopic homogeneity of bullet lead with that found in the meat, and increased blood lead concentrations in swine fed fragment-containing venison. Considering that all the carcasses we brought to the processors contained fragments (15–409 fragments counted in radiographs), the high rate of removal evident in the ground meat implies meticulous care on the part of the processors to avoid contamination, but an apparent inability of 80% of them to do so entirely. We conclude that, in a majority of cases, one or more consumers of a hunter-killed, commercially-processed deer will consume bullet lead.

We interpret the absorption of lead into the bloodstream of all four test pigs as clear evidence of the bioavailability of lead from ingested bullet fragments (Fig. 2), and we infer that human consumption of venison processed under prevailing standards of commerce results in increased blood lead concentrations. The rate of bioavailability cannot be calculated from our experiment because the exact amounts of lead in the meat packages were unknown. Rather, we directed our test at the condition experienced by human consumers of venison from rifle-killed deer of variable amounts of lead patchily distributed as fragments in ground meat or steak.

Depuration of lead in blood does not imply its excretion, but rather the sequestration of a substantial proportion in soft tissues and ultimately in bone from which it may eventually be mobilized, as during pregnancy [9] or in old age [10]. The observed elevations in blood lead concentrations, while not considered overtly toxic, would nevertheless contribute to cumulative lead burdens, and would be additive with further meals of contaminated venison. Observed blood lead concentrations of up to 3.8 µg/dL, and daily means of 2.3 and 2.2 µg/dL in the experimental animals, do approach what is considered significant with respect to adverse effects in humans by contemporary assessments [35], [36]. Whereas the CDC advisory level for intervention in individual children is 10 µg/dL in blood [37], studies now associate as little as 2 µg/dL with increased risk of cardiovascular mortality in adults [15] and impaired cognitive function in children [38]. Hauser et al. [12] detected an impact threshold of 5 µg/dL on male maturation rates, and Lanphear et al. [3] concluded that “…lead exposure in children who have maximal blood lead concentrations <7.5 µg/dL is associated with intellectual deficits.” These latter values would appear attainable with the repeated consumption of venison possible among deer hunting families, especially those incurring additional exposure from other sources.

Factors that may influence dietary lead exposure from spent lead bullets include the frequency and amount of venison consumption, degree of bullet fragmentation, anatomical path of the bullet, the care with which meat surrounding the bullet wound is removed, and any acidic treatments of the meat that would dissolve lead, i.e., coating the hanging carcass with vinegar or the use of acidic marinades in cooking. Exposure to lead from spent bullets is easily preventable if health-minded hunters use lead-free copper bullets now widely available and generally regarded as fully comparable to lead-based bullets for use in hunting [39]. The potential for toxic exposure to copper from these bullets is presumably insignificant because little or no fragmentation occurs [28], and there is no meat wastage from having to discard tissue suspected of contamination.

Fragmenting lead bullets have been in use for hunting since the early 1900s [40]. Although hunter numbers have diminished slightly in recent years, there were 10.7 million big game hunters in the United States in 2006, the majority of whom still use lead-based bullets [31], [41]. Many state wildlife agencies annually issue multiple deer harvest permits to individuals, effectively offering venison as a year-round protein staple for some families; game meat is the principal source of protein for a considerable proportion of Alaska's population [42]. Hunter-donated venison to food pantries and shelters for low income families in most states produced an estimated minimum of 9 million venison meals associated with the 2007/08 hunting season [33]. With these concerns, we anticipate that health sciences will further examine the bioavailability of lead from bullets and shot, the epidemiology of exposure, and the possible consequences among hunters, their families, and others who consume venison.
